# Intensity of physical activity and cardiovascular and all-cause mortality in diabetic patients: A cohort study (NHANES 1999–2018)

**DOI:** 10.1097/MD.0000000000048899

**Published:** 2026-05-22

**Authors:** Lingling Sun, Xiaowei Liu, Zhi Zhang, Hongfeng Jin, Lijiang Tang, Changqing Du, Yali Wang

**Affiliations:** a Department of Geriatrics, Ningbo No. 2 Hospital, Wenzhou Medical University, Ningbo, Zhejiang, PR China; b Department of Cardiology, Zhejiang Hospital, Hangzhou, Zhejiang, PR China; c School of Pharmaceutical Science, Zhejiang Chinese Medical University, Hangzhou, Zhejiang, PR China.

**Keywords:** all-cause mortality, cardiovascular outcomes, diabetes, physical activity, secondary prevention

## Abstract

Evidence guiding physical activity (PA) recommendations for individuals with diabetes, particularly those engaging in high-intensity activity, remains limited. Here, the associations between different levels of PA and mortality risk were investigated among adults with clinically diagnosed diabetes. Data from the National Health and Nutrition Examination Survey collected between 1999 and 2018 were analyzed. Adults aged 18 to 85 years with diabetes were categorized according to self-reported PA into sedentary (n = 2069), moderate-intensity (n = 498), or vigorous-intensity (n = 1661) groups. We applied Cox proportional hazards regression to assess associations between PA levels and all-cause mortality (ACM), cardiovascular mortality, and cardiocerebrovascular mortality. Overall, 4228 individuals were enrolled (mean age, 57.97 years; 53.73% male). The median follow-up period for the 3 groups was 5.25 years, 6.58 years, and 6.17 years, respectively. Over the follow-up period, 886 (17.73%) deaths from all causes and 265 (5.52%) cardiovascular deaths were recorded. The highest mortality rates were observed in the sedentary group (25.40% ACM, 8.18% cardiovascular mortality, and 9.56% cardiocerebrovascular mortality). Compared with moderate-intensity PA, the ACM risk in the sedentary group was significantly increased (hazard ratio = 1.58, 95% confidence interval: 1.23–2.02, *P* < .01), whereas the mortality risk in the vigorous-intensity PA group did not differ significantly from the moderate-intensity group (hazard ratio = 1.07, 95% confidence interval: 0.82–1.40, *P* > .05). These results indicate that the moderate-intensity group had the lowest mortality risk, whereas the sedentary group had the highest; however, vigorous-intensity PA did not further significantly reduce mortality risk. These patterns were consistent across major demographic subgroups. Moderate or vigorous PA was associated with lower all-cause, cardiovascular, and cerebrovascular mortality compared with sedentary behavior. However, after full adjustment, these associations weakened, with no significant reduction in mortality risk for vigorous activity, indicating similar survival benefits for different exercise intensities. Future research should explore the differential effects of various exercise types on mortality risk in diabetic patients to support more targeted clinical interventions.

## 1. Introduction

Diabetes mellitus (DM) is among the most prevalent noninfectious diseases worldwide and is a major contributor to global morbidity and mortality. The past 3 decades have seen an almost fourfold increase in the number of individuals living with DM. Both the onset and progression of DM are strongly affected by lifestyle factors, particularly unhealthy dietary patterns and insufficient physical activity (PA). In the overall population, higher levels of PA are associated with a reduced cardiovascular mortality (CVM) risk compared with sedentary behavior. Previous studies indicate that regular exercise contributes to improved clinical outcomes in individuals with DM.^[1–3]^

Although many studies have explored the impact of PA on CVM risk in the general population, high-quality evidence on the relationship between different intensities of PA and mortality risk in the diabetic population remains limited. Previous studies have mainly focused on short-term outcomes or specific types of PA, lacking systematic analysis of long-term effects, especially regarding the impact of vigorous-intensity exercise on mortality in diabetic patients. Additionally, there are methodological shortcomings in terms of sample selection, statistical methods, and the quantification of PA, which affect the generalizability of the findings. According to recommendations issued by the WHO,^[4]^ the risk of premature death can be reduced in adults by undertaking a minimum of 150 minutes per week of moderate-intensity PA (MPA; 3–5.9 metabolic equivalent tasks), 75 minutes per week of vigorous-intensity physical activity (VPA; ≥6 metabolic equivalent tasks), or combinations thereof, with 1 minute of VPA considered comparable to 2 minutes of MPA. Consistent with these guidelines, current public health strategies emphasize maintaining an active lifestyle to mitigate DM-related complications and mortality risk.^[5]^ However, despite existing public health guidelines for PA in individuals with diagnosed diabetes, the specific impact of PA intensity on mortality risk has not been thoroughly studied. This study, based on the National Health and Nutrition Examination Survey (NHANES) data from 1999 to 2018, aims to quantify the long-term effects of PA on all-cause mortality (ACM) and CVM risk in diabetic patients, filling this research gap and further clarifying the relationship between PA intensity and mortality risk.

Hypothesis: We hypothesize that MPA will significantly reduce both ACM and CVM in diabetic patients, while VPA will not lead to a further significant reduction in mortality risk compared with moderate-intensity activity.

## 2. Materials and methods

### 2.1. Participants

The NHANES (https://www.cdc.gov/nchs/nhanes/) is a program consisting of nationally representative cross-sectional assessments of health and nutrition in the US population. Here, data from 10 consecutive biennial NHANES survey cycles conducted between 1999 and 2018 were analyzed. Participants were selected through a complex, multistage, stratified probability sampling strategy, and data were collected in 2-year survey waves.

**Figure 1.**
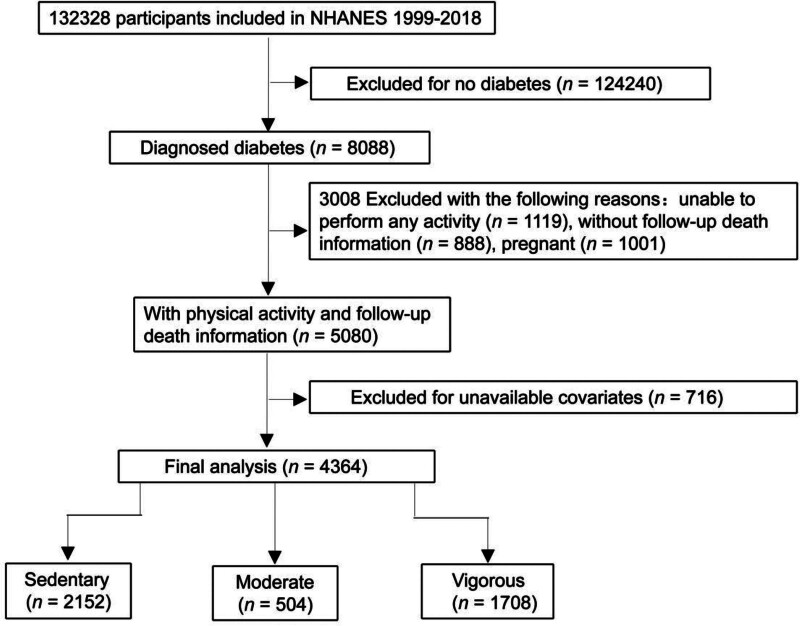
Flowchart of the study selection in this study. NHANES = National Health and Nutrition Examination Survey.

Participants were eligible for inclusion if they reported a physician diagnosis of DM and were at least 18 years old. The selection process is summarized in Figure 1. Among 132,328 NHANES respondents, 124,240 individuals without diabetes were excluded. Additional exclusions were applied to participants lacking PA data (n = 1119), mortality follow-up information (n = 888), complete covariate data (n = 716), or pediatric participants (n = 1001). After all exclusions, 4364 individuals were included in the final analysis. Based on self-reported PA intensity, participants were classified into 3 groups: sedentary (n = 2152), MPA (n = 504), and VPA (n = 1708).

### 2.2. Data

NHANES data were obtained using standardized questionnaires administered in-home interviews, complemented by clinical and laboratory assessments performed in mobile centers. The information collected included demographics (age, sex, race/ethnicity, educational attainment, health insurance coverage, socioeconomic status, and smoking status), medical history (including comorbid conditions), physical measurements (body mass index [BMI] and blood pressure [BP]), and laboratory measurements (total cholesterol, low-density lipoprotein cholesterol, and high-density lipoprotein cholesterol). Drug use information was collected through the drug survey section of NHANES. Participants completed a drug use questionnaire during face-to-face interviews, providing information on the use of all medications in the past 30 days, especially the use of diabetes treatment drugs, including metformin, sulfonylureas, glucagon-like peptide-1 receptor agonists, dipeptidyl peptidase-4 inhibitors, and sodium-glucose cotransporter 2 inhibitors. During the study, people who were taking prescription drugs were asked to bring the original packages of all the diabetes treatment drugs they were using so that the researchers could verify them. If researchers were unable to obtain the medicine boxes, they recorded the names, doses, frequency of use, and start times of the drugs reported orally by participants. All participants provided written informed consent before participation. This study was conducted in accordance with the Declaration of Helsinki; the NHANES project was approved by the National Center for Health Statistics Research Ethics Review Board, and additional ethical approval was not required for this secondary analysis of deidentified public data.

### 2.3. Parameter definitions

Hypertension was characterized as an average systolic BP of 130 mm Hg or more and/or an average diastolic BP of 80 mm Hg or more in adults with DM or current use of antihypertensive agents.^[6]^ Coronary heart disease was identified based on diagnoses of myocardial infarction, angina pectoris, or coronary artery disease.

DM was defined according to established clinical criteria, including fasting plasma glucose ≥126 mg/dL, random plasma glucose ≥200 mg/dL, or a glycated hemoglobin value ≥6.5%.^[7]^ Individuals receiving insulin or oral hypoglycemic therapy, or those with a physician-reported diagnosis of DM, were also classified as having DM.

Renal function was assessed using the estimated glomerular filtration rate, calculated with the Modification of Diet in Renal Disease equation:


$$\begin{array}{l} eGFR=186\times {\left(serum\;creatinine\right)}^{-1.154}\times {\left(age\right)}^{-0.203}\times 1.212\left(if\;Black\right)\times 0.742\left(if\;female\right) \end{array}$$

Chronic kidney disease represented an estimated glomerular filtration rate <60 mL/min/1.73 m^2^.

A diagnosis of metabolic syndrome required ≥3 of the following^[8]^: raised BP (systolic BP > 130 mm Hg or diastolic BP > 85 mm Hg, or ongoing treatment), decreased high-density lipoprotein cholesterol (<40 mg/dL in men or <50 mg/dL in women), raised fasting triglyceride levels (>150 mg/dL), impaired fasting glucose (100–125 mg/dL), or larger waist circumference (>102 cm in men or >88 cm in women).

Mortality data, including causes of death, were adjudicated by trained personnel. Causes of death were documented on official death certificates by qualified certifiers, such as physicians or medical examiners. In the NHANES dataset, deaths attributed to stroke were categorized as cerebrovascular disorders, whereas heart-related deaths were grouped under diseases of the heart. These encompassed ischemic, hypertensive, and chronic rheumatic heart disease, as well as heart failure, acute myocarditis, endocarditis, pericardial disorders, and related cardiac conditions.

According to their PA levels, patients were classified into 3 groups: the sedentary group, consisting of patients who engaged in no PA; the MPA group, comprising patients who performed MPA for ≥30 minutes per session on ≥5 days per week; and the VPA group, including patients who participated in VPA at least once weekly, with each session lasting ≥20 minutes. VPA was defined as exercise that causes a marked increase in breathing and heart rate, whereas MPA was defined as that resulting in a slight elevation in breathing or heart rate.^[9]^

### 2.4. Statistical analysis

The baseline information on the participants was summarized by PA categories and further grouped by age, sex, ethnicity/race, smoking status, educational level (associate degree or above, graduation from high school, or less than high school), socioeconomic status (annual household income <$35,000, $35,000–$75,000, and >$75,000), insurance coverage (uninsured or insured), BMI, BP, lipid profile, mortality, medicine use, and comorbidity status.

Continuous variables were compared using Kruskal–Wallis tests and are shown as mean ± standard deviation, whereas categorical variables were compared using chi-square tests and are presented as frequencies with percentages. A weighted Cox proportional hazards regression model was used for analysis to estimate the hazard ratios (HR) and 95% confidence intervals (CI) of the associations between PA levels and death outcomes (including death from cardiometabolic diseases, death from cardiovascular and cerebrovascular diseases, and all-cause death). All models incorporated the NHANES survey weights and adjusted for the stratification and clustering variables of the complex sampling design to ensure that the results were nationally representative.

The constructed regression models were Model I (unadjusted), Model II (adjusted for age, race, and sex), and Model III (additional adjustments for gender, race, age, socioeconomic status, education status, current health insurance status, and smoking). Subgroup analyses were performed using a weighted Cox proportional hazards regression model. The Wald test was used for binary variables, and the likelihood ratio test was used for those with multiple categories to assess subgroup interaction significance. Two-sided *P*-values of <.05 were considered statistically significant. All analyses were performed using SAS software version 9.4 (SAS Institute Inc., Cary).

## 3. Results

Figure 1 outlines the study selection process and censoring criteria. From the 132,328 NHANES participants surveyed between 1999 and 2018, 7988 individuals reported a history of DM. After excluding participants without PA data, mortality follow-up, or complete covariate information, 4974 adults were included in the final analysis. Of these, 2069 were classified as sedentary, 498 engaged in MPA, and 1661 reported VPA.

Baseline demographic and clinical information stratified by PA level is detailed in Table 1. The mean age of the cohort was 57.97 (95% CI: 57.31–58.62) years, and 46% were women. Higher PA levels were associated with younger age, as well as lower resting BP and higher baseline low-density lipoprotein cholesterol and total cholesterol, compared with sedentary individuals. Total cholesterol and low-density lipoprotein cholesterol levels were higher in more active participants, whereas high-density lipoprotein cholesterol levels were comparable among groups. Elevated PA levels were more frequently observed among men, non-Hispanic Black participants, and those with higher educational attainment and household income. Approximately half of the cohort reported metformin use. Sedentary participants had a significantly higher prevalence of obesity, chronic kidney disease, hypertension, coronary heart disease, metabolic syndrome, stroke, and heart failure.

**Table 1:** Baseline characteristics of participants in the final analytic sample (n = 4228) by physical activity levels: sedentary, moderate, and vigorous.

Characteristics	Overall (n* *= 4228)	Physical activity			*P* value
		Sedentary (n = 2069)	Moderate (n* *= 498)	Vigorous (n = 1661)	
Age, weighted mean (95% CI)	57.97 (57.31, 58.62)	64.24 (61.80, 63.19)	58.83 (57.42, 60.24)	53.52 (52.22, 54.81)	<.0001
Gender, weighted n (%)					<.0001
Male	2282 (53.73)	992 (46.96)	260 (50.52)	1030 (63.45)	
Female	1946 (46.27)	1077 (53.04)	238 (49.48)	631 (36.55)	
Race, weighted n (%)					.002
Non-Hispanic White	1543 (69.01)	866 (72.90)	189 (74.84)	488 (61.89)	
Hispanic	1102 (15.34)	424 (11.02)	135 (13.65)	543 (21.60)	
Non-Hispanic Black	1111 (15.65)	582 (16.07)	100 (11.51)	429 (16.50)	
Socioeconomic status, weighted n (%)					.07
Low	1820 (37.55)	968 (40.90)	210 (32.47)	642 (34.57)	
Middle	1316 (36.03)	658 (33.68)	150 (38.04)	508 (38.72)	
High	698 (26.42)	334 (25.42)	92 (29.49)	272 (26.72)	
Education status, weighted n (%)					.08
<High school	1318 (21.61)	690 (23.94)	138 (18.62)	490 (19.63)	
High school diploma	965 (24.81)	471 (24.14)	124 (30.41)	370 (23.75)	
AA or higher	1861 (53.58)	891 (51.92)	213 (50.97)	757 (56.62)	
Current health insurance status, weighted n (%)					<.0001
Uninsured	508 (9.96)	179 (6.75)	60 (10.29)	269 (14.35)	
Insured	3587 (90.04)	1883 (93.25)	424 (89.71)	1280 (85.65)	
Smoking, weighted n (%)					.03
Never smoker	2031 (47.11)	952 (45.78)	248 (46.41)	831 (49.04)	
Current smoker	839 (20.48)	396 (19.05)	84 (17.51)	359 (23.32)	
Post-smoker	1358 (32.41)	721 (35.16)	166 (36.09)	471 (27.64)	
Body mass index (kg/m^2^), weighted n (%)					.01
Normal	542 (11.11)	241 (10.00)	70 (12.37)	231 (12.04)	
Overweight	1191 (26.43)	513 (23.14)	156 (27.75)	522 (30.05)	
Obesity	2343 (62.47)	1212 (66.85)	260 (59.88)	871 (57.90)	
Lipids (mg/dL), weighted mean (95% CI)					
HDL-C	47.98 (47.24–48.73)	47.08 (46.29–47.86)	46.98 (45.45–48.50)	47.05 (45.73–48.36)	.18
TC	183.54 (181.31–185.77)	179.07 (176.17–181.97)	179.29 (174.02–184.55)	184.75 (181.02–188.48)	<.0001
LDL-C	102.93 (100.72–105.14)	97.83 (94.70–100.97)	100.71 (94.83–106.59)	108.11 (103.58–112.64)	<.0001
Blood pressure (mm Hg), weighted mean (95% CI)					
SBP	130.39 (129.60–131.17)	131.07 (129.87–132.27)	128.22 (125.89–130.56)	128.04 (126.83–129.26)	.0003
DBP	69.25 (68.67–69.84)	67.73 (66.86–68.59)	68.20 (66.50–69.90)	71.46 (70.28–72.65)	<.0001
Medications, weighted n (%)					
Metformin	2106 (50.30)	1016 (51.51)	251 (53.42)	839 (47.68)	.03
Sulfonylureas	942 (22.10)	485 (22.47)	101 (21.11)	356 (21.97)	.18
Glinides	38 (0.95)	23 (1.13)	6 (0.87)	9 (0.74)	.12
Alpha-glucosidase inhibitor	17 (0.38)	7 (0.30)	3 (0.53)	7 (0.43)	.38
Thiazolidinedione	301 (7.26)	156 (7.44)	42 (10.27)	103 (5.98)	.04
DPP-4i	330 (8.48)	186 (9.22)	34 (6.98)	110 (8.05)	.06
SGLT2i	47 (1.43)	21 (0.94)	9 (3.04)	17 (1.50)	.17
GLP-1RA	86 (2.63)	37 (2.22)	14 (3.91)	35 (2.72)	.23
Insulin	998 (24.20)	553 (26.81)	106 (19.26)	339 (22.60)	<.0001
Comorbidities, weighted n (%)					
MS	1098 (75.80)	577 (84.16)	146 (77.87)	375 (63.24)	<.0001
Central obesity	2990 (81.59)	1501 (87.60)	358 (81.12)	1131 (74.71)	.005
CHD	799 (18.51)	477 (22.18)	98 (20.94)	224 (12.93)	<.0001
Stroke	392 (8.44)	269 (11.89)	32 (4.77)	91 (5.25)	.001
HF	424 (9.12)	288 (12.40)	38 (7.70)	98 (5.36)	<.0001
HTN	3283 (76.49)	1715 (83.17)	375 (76.24)	1193 (68.08)	<.0001
CKD	752 (19.80)	514 (27.14)	72 (15.31)	166 (10.99)	<.0001

Data are presented as weighted n (%) or weighted mean (95% confidence interval).

Socioeconomic status: low, <$35,000; middle, $35,000 to $75,000; high, >$75,000.

Body mass index (BMI): normal, 18.5 < BMI ≤ 25; overweight, 25 < BMI ≤ 30; obesity, BMI > 30.

Central obesity: waist circumference >102 cm for men or >88 cm for women.

AA = associate degree, CHD = coronary heart disease, CI = confidence interval, CKD = chronic kidney disease, DBP = diastolic blood pressure, DPP-4i = dipeptidyl peptidase-4 inhibitor, GLP-1RA = glucagon‑like peptide‑1 receptor agonist, HDL-C = high-density lipoprotein cholesterol, HF = heart failure, HTN = hypertension, LDL-C = low-density lipoprotein cholesterol, MS = metabolic syndrome, SBP = systolic blood pressure, SGLT2i = sodium-glucose cotransporter 2 inhibitor, TC = total cholesterol.

**Figure 2.**
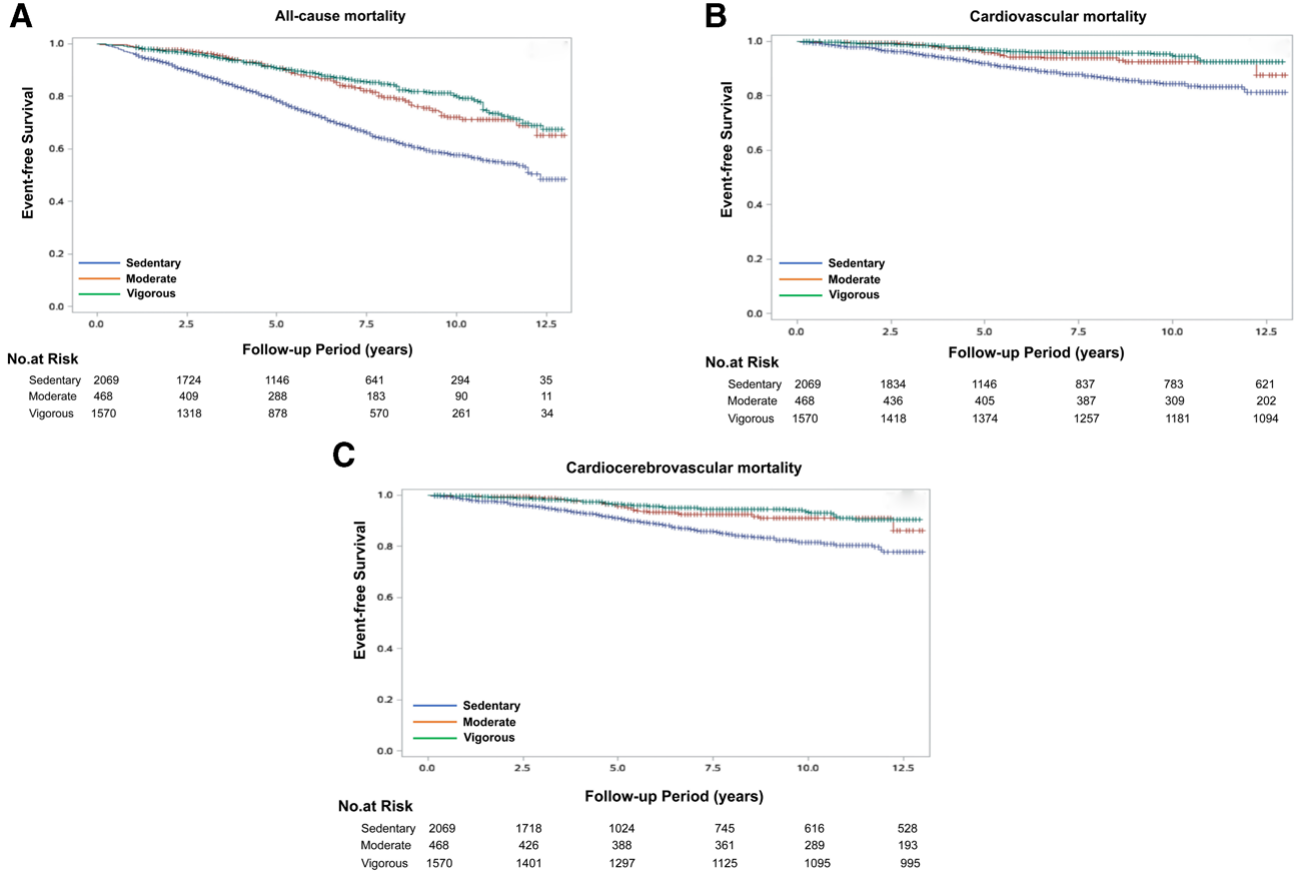
Survival curves for all-cause mortality (A), cardiovascular mortality (B), and cardiocerebrovascular mortality (C) across sedentary, moderate, and vigorous physical activity levels. Sedentary behavior is associated with higher mortality risk, while moderate and vigorous exercise show similar protective effects. The median follow-up time was 5.25 years for the sedentary group, 6.50 years for the moderate physical activity group, and 5.67 years for the vigorous physical activity group. Number-at-risk tables are presented below each panel.

Mortality outcomes according to PA category are summarized in Tables 2 and 3. During follow-up, a total of 886 deaths were documented, including 265 cardiovascular and 317 cardiocerebrovascular deaths. Sedentary individuals had markedly higher rates of CVM (8.18%), cardiocerebrovascular mortality (CCVM; 9.56%), and ACM (25.40%) than those in the moderate or vigorous PA groups (all *P* < .0001). Although mortality rates were higher in the VPA group than in the MPA group, these differences did not reach statistical significance. Figure 2 visually presents the survival curves showing the impact of sedentary behavior, MPA, and VPA on ACM, CVM, and CCVM. The survival rate of the sedentary group was significantly lower than that of the exercise groups, indicating that sedentary behavior is associated with a higher risk of mortality. The median follow-up time was 5.25 years for the sedentary group, 6.50 years for the MPA group, and 5.67 years for the VPA group. Moreover, there was no significant difference between the MPA and VPA groups, suggesting that the impact of different exercise intensities on mortality risk is similar.

**Table 2:** Association between physical activity intensity and mortality in diabetic patients: all-cause, cardiovascular, and cardiocerebrovascular mortality by physical activity levels.

Characteristics	Overall (n* *= 4228)	Physical activity			*P* value	*P* value*
		Sedentary (n = 2069)	Moderate (n* *= 498)	Vigorous (n = 1661)		
Person-years	23,367	11,746	3197	8424		
Mortality, weighted n (%)						
All-cause	886 (17.73)	596 (25.40)	78 (12.55)	212 (9.77)	<.0001	.06
Cardiovascular	265 (5.52)	187 (8.18)	23 (4.03)	55 (2.64)	<.0001	.09
Cardiocerebrovascular	317 (6.56)	220 (9.56)	27 (4.32)	70 (3.50)	<.0001	.24

Data are presented as weighted n (%).

* Comparison of mortality between moderate and vigorous groups.

**Table 3:** Hazard ratio for mortality in diabetic patients by physical activity intensity: unadjusted and adjusted for confounding factors.

Characteristics	Physical activity			*P* value
	Sedentary (n = 2069)	Moderate (n* *= 498)	Vigorous (n = 1661)	
All-cause mortality				
Event, weighted n (%)	596 (25.40)	78 (12.55)	212 (9.77)	<.0001
Unadjusted HR (95% CI)	1.76 (1.40–2.22)**	References	0.81 (0.63–1.05)	–
Adjusted HR (95% CI)‡	1.58 (1.23–2.02)**	References	1.07 (0.82–1.40)	–
Adjusted HR (95% CI)§	1.57 (1.21–2.02)**	References	0.89 (0.66–1.19)	–
Cardiovascular mortality				
Event, weighted n (%)	187 (8.18)	23 (4.03)	55 (2.64)	<.0001
Unadjusted HR (95% CI)	1.90 (1.25–2.91)**	References	0.69 (0.43–1.11)	–
Adjusted HR (95% CI)‡	1.57 (1.02–2.43)*	References	0.86 (0.53–1.41)	–
Adjusted HR (95% CI)§	1.52 (0.97–2.40)	References	0.68 (0.40–1.16)	–
Cardiocerebrovascular mortality				
Event, weighted n (%)	220 (9.56)	27 (4.32)	70 (3.50)	<.0001
Unadjusted HR (95% CI)	1.90 (1.29–2.82)**	References	0.75 (0.49–1.17)	–
Adjusted HR (95% CI)‡	1.55 (1.04–2.32)**	References	0.95 (0.61–1.48)	–
Adjusted HR (95% CI)§	1.49 (0.99–2.27)	References	0.74 (0.45–1.20)	–

CI = confidence interval, HR = hazard ratio.

‡ Adjusted for age, gender, and race.

§ Adjusted for gender, race, age, socioeconomic status, education status, current health insurance status, and smoking.

* *P* < .05.

** *P* P < .01 versus moderate physical activity group.

The results of the Cox regression analysis (Table 3) show that, in Models I and II, sedentary behavior is significantly associated with a higher risk of mortality compared with the MPA group. In the fully adjusted model (Model III), the risk of ACM is significantly increased in the sedentary group (HR = 1.57, 95% CI: 1.21–2.02), while no statistically significant difference is observed between the VPA group and the MPA group (HR = 0.89, 95% CI: 0.66–1.19). A similar trend is seen in CVM and CCVM, where sedentary behavior still shows an increased risk in the unadjusted models, but the association weakens and becomes nonsignificant in the fully adjusted models (ACM: HR = 1.52, 95% CI: 0.97–2.40; CCVM: HR = 1.49, 95% CI: 0.99–2.27). Overall, sedentary behavior is associated with an increased risk of mortality, but no significant differences are observed between different exercise intensities.

Subgroup analyses (Table 4) revealed consistent associations between sedentary lifestyles and higher ACM across nearly all strata, including sex, race/ethnicity, socioeconomic status, educational attainment, insurance coverage, BMI categories, and comorbidity status (almost all *P* < .05). VPA was associated with reduced ACM across multiple demographic and clinical subgroups, including genders, Hispanic and non-Hispanic Black groups, various socioeconomic groups, individuals with different educational levels and insured groups, various weight groups, and comorbidities; however, it did not confer additional benefit beyond MPA. Consistent findings were observed for CVM and CCVM outcomes (Tables 5 and 6).

**Table 4:** Subgroup analyses of the association between physical activity intensity and all-cause mortality in diabetic patients.

Subgroup	All-cause mortality					
	Sedentary vs moderate			Vigorous vs moderate		
	HR	95% CI	*P*	HR	95% CI	*P*
Gender						
Male	1.72	1.27–2.33	.0004	0.81	0.59–1.12	.21
Female	1.85	1.29–2.66	.0008	0.72	0.47–1.10	.12
Race						
Non-Hispanic White	1.90	1.35–2.67	.0002	1.12	0.77–1.64	.55
Hispanic	1.42	0.85–2.36	.18	0.73	0.43–1.24	.25
Non-Hispanic Black	1.80	1.08–2.99	.02	0.76	0.43–1.32	.33
Socioeconomic status						
Low	1.65	1.22–2.23	.001	0.70	0.50–0.98	.04
Middle	2.06	1.28–3.30	.003	0.71	0.42–1.20	.20
High	2.41	1.04–5.60	.04	0.74	0.29–1.94	.54
Education status						
<High school	1.51	1.06–2.15	.02	0.57	0.38–0.85	.006
High school diploma	1.69	1.07–2.68	.02	0.93	0.56–1.53	.77
AA or higher	1.83	1.22–2.75	.004	0.97	0.63–1.51	.90
Current health insurance status						
Uninsured	0.97	0.39–2.44	.95	0.73	0.29–1.81	.50
Insured	1.92	1.50–2.45	<.0001	0.72	0.54–0.95	.02
Body mass index (kg/m^2^)						
Normal	2.02	1.16–3.53	.01	0.69	0.37–1.28	.24
Overweight	1.60	1.05–2.42	.03	1.01	0.65–1.56	.98
Obesity	1.56	1.11–2.18	.01	0.70	0.48–1.02	.06
Comorbidities						
MS						
No	3.21	1.38–7.47	.007	0.87	0.35–2.19	.77
Yes	1.61	1.07–2.41	.02	0.85	0.54–1.34	.48
Central obesity						
No	1.32	1.01–1.73	.045	0.76	0.56–1.02	.07
Yes	2.72	1.58–4.67	.0003	0.91	0.51–1.61	.74
CHD						
No	1.65	1.24–2.19	.0006	0.87	0.64–1.18	.35
Yes	1.75	1.18–2.61	.006	0.73	0.45–1.16	.18
Stroke						
No	1.57	1.23–2.00	.0003	0.77	0.59–1.00	.05
Yes	2.42	1.13–5.18	.02	1.19	0.51–2.80	.69
HF						
No	1.60	1.24–2.07	.0003	0.84	0.64–1.11	.23
Yes	1.74	1.01–3.01	.046	0.61	0.31–1.18	.14
HTN						
No	2.05	2.17–3.59	.01	0.60	0.32–1.12	.11
Yes	1.68	1.30–2.16	<.0001	0.88	0.67–1.16	.36
CKD						
No	1.63	1.17–2.26	.004	0.69	0.48–0.99	.04
Yes	1.79	1.13–2.82	.01	0.78	0.46–1.35	.38

Socioeconomic status: low, <$35,000; middle, $35,000 to $75,000; high, >$75,000.

Body mass index (BMI): normal, 18.5 < BMI ≤ 25; overweight, 25 < BMI ≤ 30; obesity, BMI > 30.

Central obesity: waist circumference >102 cm for men or >88 cm for women.

AA = associate degree, CHD = coronary heart disease, CI = confidence interval, CKD = chronic kidney disease, HF = heart failure, HR = hazard ratio, HTN = hypertension, MS = metabolic syndrome.

**Table 5:** Subgroup analyses of the association between physical activity intensity and cardiovascular mortality in diabetic patients.

Subgroup	Cardiovascular mortality					
	Sedentary vs moderate			Vigorous vs moderate		
	HR	95% CI	*P*	HR	95% CI	*P*
Gender						
Male	1.72	1.27–2.33	.0004	0.81	0.59–1.12	.21
Female	1.85	1.29–2.66	.0008	0.72	0.47–1.10	.12
Race						
Non-Hispanic White	1.91	1.06–3.47	.03	1.04	0.53–2.04	.90
Hispanic	1.15	0.47–2.82	.76	0.56	0.22–1.46	.24
Non-Hispanic Black	1.91	0.77–4.75	.16	0.48	0.17–1.38	.17
Socioeconomic status						
Low	1.61	0.96–2.71	.07	0.50	0.27–0.93	.03
Middle	6.51	1.59–26.62	.009	1.50	0.33–6.76	.60
High	1.19	0.34–4.15	.78	0.32	0.06–1.57	.16
Education status						
<High school	1.58	0.84–2.94	.15	0.46	0.22–0.96	.04
High school diploma	2.20	0.87–5.56	.09	1.20	0.44–3.22	.72
AA or higher	1.78	0.85–3.71	.13	0.66	0.29–1.51	.32
Current health insurance status						
Uninsured	1.22	0.14–10.91	.86	0.82	0.09–7.35	.86
Insured	2.07	1.32–3.25	.002	0.51	0.30–0.88	.02
Body mass index (kg/m^2^)						
Normal	1.10	0.45–2.69	.84	0.47	0.17–1.30	.15
Overweight	2.03	0.92–4.49	.08	0.89	0.38–2.11	.80
Obesity	1.98	1.03–3.80	.04	0.72	0.34–1.50	.37
Comorbidities						
MS						
No	1.98	0.58–6.79	.28	0.39	0.09–1.73	.21
Yes	1.47	0.73–2.98	.28	0.67	0.30–1.52	.34
Central obesity						
No	1.63	0.96–2.74	.07	0.72	0.40–1.29	.27
Yes	1.69	0.74–3.85	.21	0.64	0.27–1.54	.32
CHD						
No	1.71	0.98–2.98	.06	0.83	0.46–1.52	.55
Yes	1.93	1.01–3.70	.047	0.52	0.23–1.19	.12
Stroke						
No	1.78	1.13–2.80	.01	0.70	0.42–1.17	.17
Yes	1.80	0.56–5.82	.33	0.53	0.12–2.36	.40
HF						
No	1.74	1.05–2.89	.03	0.80	0.46–1.40	.44
Yes	1.55	0.71–3.36	.27	0.42	0.15–1.16	.09
HTN						
No	1.55	0.59–4.04	.37	0.31	0.10–1.02	.05
Yes	1.94	1.21–3.11	.006	0.81	0.48–1.38	.44
CKD						
No	1.37	0.79–2.37	.27	0.36	0.18–0.70	.003
Yes	2.03	0.89–4.66	.09	0.96	0.37–2.49	.93

Socioeconomic status: low, <$35,000; middle, $35,000 to $75,000; high, >$75,000.

Body mass index (BMI): normal, 18.5 < BMI ≤ 25; overweight, 25 < BMI ≤ 30; obesity, BMI > 30.

Central obesity: waist circumference >102 cm for men or >88 cm for women.

AA = associate degree, CHD = coronary heart disease, CI = confidence interval, CKD = chronic kidney disease, HF = heart failure, HR = hazard ratio, HTN = hypertension, MS = metabolic syndrome.

**Table 6:** Subgroup analyses of the association between physical activity intensity and cardiocerebrovascular mortality in diabetic patients.

Subgroup	Cardiocerebrovascular mortality					
	Sedentary vs moderate			Vigorous vs moderate		
	HR	95% CI	*P*	HR	95% CI	*P*
Gender						
Male	1.80	1.08–2.98	.02	0.81	0.47–1.39	.43
Female	2.10	1.13–3.90	.02	0.52	0.24–1.14	.10
Race						
Non-Hispanic White	1.95	1.12–3.38	.01	1.14	0.62–2.10	.68
Hispanic	1.11	0.48–2.57	.80	0.62	0.26–1.49	.29
Non-Hispanic Black	1.83	0.79–4.21	.16	0.47	0.18–1.24	.13
Socioeconomic status						
Low	1.55	0.96–2.50	.07	0.55	0.32–0.97	.04
Middle	5.08	1.60–16.14	.006	1.19	0.34–4.18	.78
High	1.70	0.51–5.72	.39	0.32	0.06–1.57	.16
Education status						
<High school	1.55	0.87–2.75	.14	0.57	0.30–1.10	.09
High school diploma	2.51	0.99–6.29	.05	1.34	0.50–3.56	.56
AA or higher	1.71	0.89–3.31	.11	0.64	0.31–1.36	.25
Current health insurance status						
Uninsured	1.23	0.14–11.01	.85	1.45	0.18–11.80	.73
Insured	2.03	1.35–3.07	.0007	0.56	0.34–0.91	.02
Body mass index (kg/m^2^)						
Normal	1.28	0.53–3.10	.58	0.48	0.17–1.31	.15
Overweight	2.08	0.99–4.36	.05	1.10	0.50–2.42	.81
Obesity	1.76	0.99–3.13	.05	0.69	0.36–1.32	.26
Comorbidities						
MS						
No	2.48	0.74–8.34	.14	0.68	0.18–2.62	.57
Yes	1.43	0.75–2.71	.28	0.73	0.35–1.51	.39
Central obesity						
No	1.50	0.93–2.39	.09	0.74	0.44–1.25	.26
Yes	1.86	0.82–4.20	.14	0.72	0.30–1.70	.45
CHD						
No	1.84	1.10–3.10	.02	0.95	0.55–1.66	.86
Yes	1.76	0.97–3.19	.06	0.51	0.24–1.08	.08
Stroke						
No	1.72	1.13–2.61	.01	0.75	0.48–1.19	.23
Yes	2.22	0.69–7.11	.18	0.66	0.16–2.78	.57
HF						
No	1.75	1.11–2.76	.02	0.85	0.52–1.39	.50
Yes	1.60	0.74–3.48	.23	0.47	0.18–1.27	.14
HTN						
No	1.79	0.69–4.62	.23	0.42	0.14–1.27	.12
Yes	1.87	1.22–2.88	.004	0.85	0.53–1.37	.51
CKD						
No	1.56	0.92–2.65	.10	0.54	0.30–0.99	.04
Yes	1.50	0.76–3.00	.25	0.64	0.28–1.48	.29

Socioeconomic status: low, <$35,000; middle, $35,000 to $75,000; high, >$75,000.

Body mass index (BMI): normal, 18.5 < BMI ≤ 25; overweight, 25 < BMI ≤ 30; obesity, BMI > 30.

Central obesity: waist circumference >102 cm for men or >88 cm for women.

AA = associate degree, CHD = coronary heart disease, CI = confidence interval, CKD = chronic kidney disease, HF = heart failure, HR = hazard ratio, HTN = hypertension, MS = metabolic syndrome.

## 4. Discussion

In this long-term observational study of adults with DM, engagement in PA was linked to reduced risk of CVM, CCVM, and ACM relative to complete inactivity. However, after comprehensive adjustment for demographic and clinical confounders, these associations were attenuated and no longer reached statistical significance. No meaningful difference in ACM was observed between VPA and MPA, suggesting that higher intensity may not confer additional survival benefit in this population.

PA is well established as improving glycemic control,^[10,11]^ promoting weight reduction,^[12]^ and enhancing cardiorespiratory fitness in individuals with DM.^[10,13]^ Improvements in cardiorespiratory fitness and body composition have also been linked to a lower incidence of heart failure.^[13]^ Our findings align with the guidelines of the American Diabetes Association, which emphasize the importance of regular PA as part of DM management. Sedentary individuals in this cohort tended to be obese and to have a history of smoking and multiple comorbid conditions, factors that collectively contribute to elevated mortality risk. Although PA improves insulin sensitivity, muscular strength, and aerobic capacity,^[14]^ our data indicate that only about half of adults with DM achieve recommended levels of PA, consistent with previous reports of widespread physical inactivity among individuals with type 2 DM.^[15]^

Previous studies have consistently demonstrated that moderate-to-high levels of aerobic exercise are related to significant reductions in both CVM and ACM among patients with DM.^[16–18]^ Thus, structured exercise is increasingly recognized as a key component of comprehensive DM care.^[19]^ Beyond metabolic control, PA enhances quality of life by modulating skeletal muscle enzymes and signaling pathways involved in glucose uptake and insulin action,^[20]^ and may slow mobility decline in overweight individuals with DM.^[21]^ Current guidelines recommend a combination of aerobic and resistance training performed on a regular basis.

The American Diabetes Association further recommends minimizing sedentary time through light activities, such as walking, in addition to MPA or VPA.^[22]^ Evidence from the Whitehall II investigation indicates that even limited amounts of moderate-to-vigorous PA can reduce ACM risk in individuals with type 2 DM.^[23]^ A US cohort study also reached a similar conclusion, that moderate-to-vigorous PA is associated with a lower risk of diabetes-related death.^[24]^ These results suggest that any level of PA, irrespective of intensity, may be an effective strategy to promote long-term health among people with DM.^[25]^ A meta-analysis supports this view. The study shows that an emphasis on “even a small increase in activity level” is also associated with better long-term health indicators.^[26]^

In this extended follow-up analysis of a large diabetic cohort, engagement in moderate-to-vigorous PA was linked to markedly reduced risks of ACM, CVM, and CCVM compared with complete inactivity. Individuals with sedentary lifestyles consistently exhibited the highest mortality risks across all outcomes, with more than a twofold increase in CVM, CCVM, and ACM. However, there was no significant difference in ACM between the vigorous-intensity and MPA groups. Although the VPA group exhibited a lower mortality rate in the unadjusted model, this difference was not statistically significant in the fully adjusted model. This indicates that both moderate and vigorous physical activities can provide survival benefits in certain cases, but their impact on mortality risk is similar, and VPA does not significantly outperform moderate-intensity activity.

Therefore, the results of this study suggest that, in diabetic patients, the effect of exercise intensity on mortality risk is consistent in the adjusted model. This finding implies that the marginal effect of exercise intensity on reducing mortality risk is small, highlighting that regular PA, regardless of intensity, remains an effective strategy to reduce mortality risk in diabetic patients. This conclusion is consistent with existing research and further emphasizes the importance of maintaining an active lifestyle for the health of diabetic patients.

Subgroup analyses further supported a consistent protective association between PA and adverse outcomes across diverse demographic and clinical strata. Previous research also demonstrates a strong inverse relationship between mortality, cardiorespiratory fitness, and PA levels in individuals with DM, with particularly pronounced risk elevations observed in those with poor fitness levels. The beneficial effects of PA are not confined to cardiovascular health but extend to broader physiological and metabolic domains.

The study has several strengths and limitations. A prominent strength is the use of NHANES data, a nationally representative dataset collected under strict quality-control protocols, ensuring robust data reliability. The substantial size of the sample and follow-up length further enhanced statistical precision and reduced recall-related bias. However, the first limitation of this study is missing data, with approximately 3724 diabetic individuals excluded due to missing data; excluding these individuals may affect the representativeness of the sample and the generalizability of the results. As multiple imputation was not used to handle missing data, future studies may consider employing more advanced data handling methods. Second, the cross-sectional nature of NHANES precludes assessment of individual-level changes in PA over time. Although extensive covariate adjustment was performed, residual confounding cannot be fully eliminated. Furthermore, reverse causation remains possible, as poorer health may predispose individuals to no PA or lower-intensity PA. Finally, variations in the length of follow-up may have influenced the estimation of long-term mortality risk.

Although previous studies have explored the relationship between physical exercise and mortality among diabetic patients, this study provides new insights by using long-term data from the NHANES database and comparing the effects of different exercise intensities on the risk of death. Although this study did not show a clear advantage of vigorous exercise over moderate-intensity exercise, we found that regular physical exercise, regardless of intensity, remains a key strategy for improving survival, overall health, and quality of life of diabetic patients. Future research can further explore the differential effects of different types of exercise (such as aerobic, resistance, and flexibility training) to provide a more comprehensive basis for clinical practice.

## 5. Conclusion

This study analyzed the relationship between PA intensity and mortality risk in diabetic patients using NHANES data. The results showed that, compared with sedentary behavior, engaging in moderate or vigorous PA was associated with lower ACM, CVM, and cerebrovascular mortality. However, after full adjustment, VPA did not significantly reduce the mortality risk, indicating that the survival benefits of different activity intensities are similar. Subgroup analyses further confirmed the protective effect of physical exercise across different demographic and clinical backgrounds. Although vigorous activity did not show additional benefits, regular PA at any level remains an effective strategy for reducing mortality risk in diabetic patients.

## Acknowledgments

We thank all the staff and reviewers who participated in the review during the preparation of this manuscript.

## Author contributions

**Data curation:** Lingling Sun, Yali Wang.

**Methodology:** Lingling Sun, Zhi Zhang.

**Software:** Xiaowei Liu.

**Visualization:** Hongfeng Jin.

**Validation:** Lijiang Tang.

**Supervision:** Changqing Du, Yali Wang.

**Writing – original draft:** Lingling Sun.

**Writing – review & editing:** Xiaowei Liu, Zhi Zhang, Yali Wang.
